# Regulation of WRKY46 Transcription Factor Function by Mitogen-Activated Protein Kinases in *Arabidopsis thaliana*

**DOI:** 10.3389/fpls.2016.00061

**Published:** 2016-02-04

**Authors:** Arsheed H. Sheikh, Lennart Eschen-Lippold, Pascal Pecher, Wolfgang Hoehenwarter, Alok K. Sinha, Dierk Scheel, Justin Lee

**Affiliations:** ^1^Department of Stress and Developmental Biology, Leibniz Institute of Plant BiochemistryHalle/Saale, Germany; ^2^National Institute of Plant Genome ResearchNew Delhi, India

**Keywords:** mitogen-activated protein kinase, WRKY transcription factors, phosphorylation, protein stability, defense, pathogen-associated molecular patterns (PAMPs)

## Abstract

Mitogen-activated protein kinase (MAPK) cascades are central signaling pathways activated in plants after sensing internal developmental and external stress cues. Knowledge about the downstream substrate proteins of MAPKs is still limited in plants. We screened *Arabidopsis* WRKY transcription factors as potential targets downstream of MAPKs, and concentrated on characterizing WRKY46 as a substrate of the MAPK, MPK3. Mass spectrometry revealed *in vitro* phosphorylation of WRKY46 at amino acid position S168 by MPK3. However, mutagenesis studies showed that a second phosphosite, S250, can also be phosphorylated. Elicitation with pathogen-associated molecular patterns (PAMPs), such as the bacterial flagellin-derived flg22 peptide led to *in vivo* destabilization of WRKY46 in *Arabidopsis* protoplasts. Mutation of either phosphorylation site reduced the PAMP-induced degradation of WRKY46. Furthermore, the protein for the double phosphosite mutant is expressed at higher levels compared to wild-type proteins or single phosphosite mutants. In line with its nuclear localization and predicted function as a transcriptional activator, overexpression of WRKY46 in protoplasts raised basal plant defense as reflected by the increase in promoter activity of the PAMP-responsive gene, *NHL10*, in a MAPK-dependent manner. Thus, MAPK-mediated regulation of WRKY46 is a mechanism to control plant defense.

## Introduction

Upon pathogen encounter, plants respond through two layers of immunity: PTI (pattern-triggered immunity) and ETI (effector-triggered immunity; Jones and Dangl, 2006). PTI is the generalized defense mechanism in which conserved pathogen-associated molecular patterns (PAMPs) like bacterial flagellin, elongation factor Tu (EF-Tu) and fungal chitin are recognized at the cell surface by specialized pattern-recognition receptors (PRRs; Boller and Felix, 2009). One of the best studied plant PAMP–PRR interactions is that of the elicitor-active 22-amino acid peptide derived from bacterial flagellin (flg22) and the flg22 receptor, FLAGELLIN-SENSING 2 (FLS2), in *Arabidopsis thaliana*. FLS2 contains an extracellular leucine-rich repeat (LRR) domain for ligand binding, a transmembrane domain and a cytoplasmic serine/threonine kinase domain (Gómez-Gómez and Boller, 2002). After activation by flg22, FLS2 interacts with BRI1-ASSOCIATED RECEPTOR KINASE 1 (BAK1) to initiate PTI (Chinchilla et al., 2007). The general cellular responses in PTI include Ca^2+^ fluxes, reactive oxygen species (ROS) production, activation of mitogen-activated protein kinases (MAPKs), transcriptional reprogramming like *PR-1* and *WRKY* gene expression and production of antimicrobial compounds like phytoalexins (Dodds and Rathjen, 2010). However, adapted pathogens are able to suppress PTI through effector molecules, which are mostly delivered into the plant cell. During plant-pathogen co-evolution, some plants have developed intracellular LRR-receptors to recognize, such effectors or the modifications they cause, and subsequently initiate the typically stronger ETI defense response (Chisholm et al., 2006). ETI usually results in localized cell death (hypersensitive response, HR) to restrict the growth of biotrophic pathogens.

Activation of MAPK signaling is vital for mounting an appropriate PTI response. A typical MAPK cascade consists of a modular complex comprising a MAPK kinase kinase (MAPKKK), phosphorylating a MAPK kinase (MAPKK), which phosphorylates a MAPK (Sinha et al., 2011; Meng and Zhang, 2013). Then, the activated MAPK specifically phosphorylates various substrate proteins, such as transcription factors, at conserved amino acid residues (S/T-P motif). After flg22 perception, two *Arabidopsis* MAPK pathways are activated. One pathway involves the MAPKKs, MKK4, and MKK5, which act upstream of the MAPKs, MPK3 and MPK6, leading to the activation of WRKY transcription factors that positively regulate defense gene expression (Asai et al., 2002). The second flg22-activated MAPK cascade is composed of MEKK1, MKK1/MKK2 (two redundant MAPKKs), and MPK4. Based on both genetic and biochemical studies, MPK4 exhibits negative control of salicylic acid (SA)-regulated plant defense (Petersen et al., 2000). A recent study identified a more complex scheme of MAPK-dependent defense gene expression. Here, MPK3 and MPK4 influenced subsets of defense-related genes both negatively and positively in their expression (Frei dit Frey et al., 2014). A fourth MAPK, MPK11, is also activated by flg22 treatment (Bethke et al., 2012) or other PAMPs (Eschen-Lippold et al., 2012).

Knowledge regarding the downstream targets of MAPKs is still fragmentary and high-throughput methods have been employed to identify the potential MAPK substrates. The various strategies used include yeast-two hybrid screens, protein microarrays and *in vitro* kinase assays. Microarray-based testing of 1690 *Arabidopsis* proteins identified 48 *in vitro* substrates for MPK3 and 39 for MPK6, with an overlapping set of 26 candidates (Feilner et al., 2005). High-density protein microarrays used to determine the phosphorylation targets of 10 different MAPKs identified 570 potential MAPK targets out of 2158 candidates (Popescu et al., 2009). Some of the well characterized MAPK targets relevant to pathogen response include the 1-aminocyclopropane-1-carboxylic acid synthase (ACS), ethylene response factor 104 (ERF104), tandem zinc finger 9 (TZF9), VirE1-interacting protein 1 (VIP1), WRKY33 transcription factor, MPK4 substrate 1 (MKS1), and several related proteins with so-called “VQ-motifs” (Meng and Zhang, 2013; Maldonado-Bonilla et al., 2014; Pecher et al., 2014).

WRKY transcription factors control transcriptional reprogramming to mediate cellular responses to diverse environmental cues (Eulgem and Somssich, 2007) and many WRKYs are targets of *Arabidopsis* MAPKs (Popescu et al., 2009). Besides regulating plant development, WRKYs are important positive and negative regulators of both PTI and ETI (van Verk et al., 2011). The defining feature of WRKYs is their DNA binding WRKY domain. The WRKY domain comprises the highly conserved WRKYGQK peptide sequence and a zinc finger motif (CX_4-7_ -CX_22-23_ -HXH/C, where X represents any amino acid and C/H are the conserved zinc-coordinating cysteine or histidine residues). This domain is responsible for binding to the W-box element with the consensus “(C/T)TGAC(T/C)” nucleotide sequence, although flanking DNA sequences appear to contribute to binding specificities (Ciolkowski et al., 2008). The 74 members of *Arabidopsis* WRKYs are divided into three groups based on the number of WRKY domains (two domains in group I, and one in groups II and III) and the arrangement of conserved cysteine/histidine residues of their zinc fingers (C–C–H–H in group I/II but C–C–H–C in group III; Rushton et al., 2010). In *Arabidopsis*, it has been shown that WRKY33 exists in nuclear complexes with MPK4 and MKS1. PAMP perception activates MPK4, leading to the nuclear dissociation of the MPK4–MKS1–WRKY33 complex and thereby releasing WRKY33 and MKS1. WRKY33 then activates expression of *PAD3* (*Phytoalexin Deficient 3*), a key enzyme for the synthesis of antimicrobial camalexin (Qiu et al., 2008). Additionally, MPK3 and MPK6 are major contributors to fungus-induced camalexin accumulation by phosphorylating WRKY33 (Mao et al., 2011). Taken together, these two studies suggest transcriptional regulation of camalexin biosynthesis gene expression through a release of WRKY33 from an inhibitory MPK4-containing protein complex and a positive effect on WRKY33 function through MPK3/6-mediated WRKY33 phosphorylation.

In the current study, 48 *Arabidopsis* WRKY proteins were investigated as *in vitro* MPK3 and MPK6 phosphorylation targets; and one member, WRKY46 was selected for further characterisation since its importance in plant pathogen response and abiotic stresses is indicated in numerous studies (van Verk et al., 2011; Moreau et al., 2012; Ding et al., 2013, 2014; Gao et al., 2013). Our findings establish WRKY46 as an MAPK phospho-target mediating plant defense responses.

## Materials and Methods

### Plant Growth Conditions and Genotypes

All plants were grown in growth chambers with a photoperiod of 8 h light (120 μmol m^-2^ s^-1^; 22°C) and 16 h dark (20°C). *Arabidopsis thaliana* ecotype Col-0 was used as wild type. The *mpk3* and *mpk6* mutants (in Col-0 background) were described previously (Wang et al., 2008; Bethke et al., 2009). *Nicotiana benthamiana* was used for *Agrobacterium tumefaciens*-mediated transient expression experiments.

### Site-Directed Mutagenesis

The *WRKY46^S168A^, WRKY46^S250A^*, and *WRKY46^S168A,S250A^* phosphorylation site variants were generated as described (Palm-Forster et al., 2012; Eschen-Lippold et al., 2014). Briefly, mutagenesis primers (as listed in **Table 1**) with a flanking *Bsa*I site were used to amplify the entire pDONR220 vector harboring *WRKY46*. After *Dpn*I digestion of the template plasmid, the amplified vector was gel-purified, digested with *Bsa*I and ligated. The mutated *WRKY46* variants were then transferred to destination vectors by standard Gateway^TM^ LR- recombination-based cloning (Invitrogen). For the phosphomimetic *WRKY46^S168D,S250D^* variant, two fragments were amplified by PCR using the primer pairs, W46-S168D_3/W46-S250D_5 and W46-S168D_5/W46-S250D_3, and *pUGW15-WRKY46* as a template. Both fragments were gel-purified and mixed and incubated in the presence of *Lgu*I and T4-DNA ligase in a PCR cycler machine (10 cycles of 10 min 37°C and 10 min 22°C).

**Table 1:** Primers used for site-directed mutagenesis.

Name	Primer^∗^	Purpose
WRKY46PSM1 F	TT GGTCTC A AGATCTTGCTCCTGCAACATCA	S250A mutation
WRKY46PSM1 R	TT GGTCTC A ATCTTCCACGAAATTTCCCAAGA	S250A mutation
WRKY46PSM2 F	TT GGTCTC A TCACAGCCCCGAAGACGACGAC	S168Amutation
WRKY46PSM2 R	TT GGTCTC A GTGATGTTGTTACAAGTGTGGTTTCC	S168A mutation
W46-S168D_5	AAAAAA GCTCTTC C ACAGATCCGAAGACGACGACG	S168D mutation
W46-S168D_3	AAAAAA GCTCTTC A TGTGATGTTGTTACAAGTGTG	S168D mutation
W46-S250D_5	AAAAAA GCTCTTC T GATCCTGCAACATCAGGGTCT	S250D mutation
W46-S250D_3	AAAAAA GCTCTTC G ATCAAGATCTTCCACGAAATT	S250D mutation

*^∗^Note that the Type IIs restriction sites (*Bsa*I or *Lgu*I) are underlined.*

### Preparation of Recombinant Proteins and *In Vitro* Phosphorylation Assay

The 48 WRKY coding sequences were cloned in-frame into expression vector pDEST-N110 (Dyson et al., 2004) to generate N-terminally His_10_-tagged recombinant proteins. The vectors were transformed into *KRX* competent cells (Promega). Cells were grown in 2xYT media (16 g L^-1^ Bacto tryptone, 10 g L^-1^ Bacto yeast extract, 5 g L^-1^ NaCl, pH 7.2) to OD_600_ of 0.5–0.6 and protein expression was induced at 24°C overnight by adding 1 mM rhamnose. Denaturing purification was performed by resuspending the pellet from 100 mL culture in 20 mL of lysis buffer (100 mM sodium phosphate, 10 mM Tris, 6 M GuHCl, pH 8) and incubated at room temperature for 30 min. After centrifugation the supernatant was incubated for 1 h with 100 μL of equilibrated Ni-NTA-Agarose beads (Thermo Scientific). The beads were washed thrice with wash buffer (100 mM sodium phosphate, 10 mM Tris, 8 M Urea, pH 6.3). The proteins were refolded on the beads as previously described (Feilner et al., 2005). Briefly, 130 μL, 260 μL and 1 mL of native buffer (10 mM Tris pH 7.5, 1 mM PMSF) was consecutively added within a period of 30 min (on ice) to reduce urea concentration to <0.5 M. The supernatant after a brief centrifugation pulse was removed and 1 mL of native buffer added and refolding of the proteins allowed to proceed for another 2 h on ice.

*In vitro* kinase assay was performed as described (Feilner et al., 2005; Pecher et al., 2014) with slight modifications. Briefly, after visual estimation (by coomassie staining) of the amount of proteins purified, 5–10 μL of the Ni-NTA resin containing the bound protein was mixed with reaction buffer to give a final volume of 15 μL containing 25 mM Tris-Cl (pH 7.5), 10 mM MgCl_2_, 1 mM DTT, 1 mM PMSF, 25 μM ATP, 1 μCi [γ-^32^P]ATP and ∼100 ng of active MAPKs. After 30 min incubation at 30°C, the reaction was stopped by addition of one volume of 2x SDS sample buffer. Samples were boiled at 95°C for 5 min and then separated on 10% SDS-PAGE gel and phosphorylation of the protein substrates visualized by phosphor-imaging (Typhoon Phosphorimaging System, GE Healthcare) after drying the gel.

### ProQ Diamond Staining of Phosphoproteins

The MPK:WRKY proteins were incubated at approximately 1:10 (w/w) ratio for 30 min as described above (except for the lack of radioactive ATP). After separation by SDS-PAGE, the gel was fixed with 50% methanol and 10% acetic acid overnight. The gel was washed with water for 30 min and stained with 3x diluted Pro-Q diamond stain (Invitrogen) in the dark for 2 h. The gel was destained four times for 30 min each with 20 % acetonitrile, 50 mM sodium-acetate (pH 4.2). The gel was washed again with water for 10 min and was scanned at 400 V using a Typhoon Scanner (GE Healthcare).

### Mass Spectrometric Analysis

Phosphorylated proteins were separated by 10% SDS-PAGE. The gel was stained with Colloidal Coomassie Blue Stain (Life Technologies) and washed with water. The corresponding gel bands were excised, destained by multiple washes with 30% acetonitrile, 100 mM NH_4_HCO_3_ and finally rinsed twice with water. After standard reduction and alkylation (Pecher et al., 2014), the proteins in the gel pieces were digested overnight at 37°C by incubation in 5% acetonitrile, 10 mM NH_4_HCO_3_ with 3 ng μL^-1^ modified trypsin (Promega). Peptides were extracted into 35% acetonitrile with 0.4% TFA, dried down in a Speed-vac and finally resuspended in 20 μL of 0.1% TFA. After 5 min of sonication in a sonifier water bath, the peptides were either frozen for storage or immediately analyzed on an LC-MS system consisting of a split-free nano-LC (Easy-nLC II, Proxeon, Thermo Scientific) coupled to a hybrid-FT-mass spectrometer (Orbitrap Velos Pro, Thermo Scientific). Additional targeted LC-MS measurements of selected phosphopeptides were performed using an inclusion list in a data dependent acquisition (DDA) scan method. Peptide fragmentation was achieved using CID with MSA (multi-stage activation). Proteins were identified and phosphorylation sites were identified using Mascot V.2.3.02 and the Phospho RS module in Proteome Discoverer v1.3.

### *In Silico* Prediction of MAPK Targets

*In silico* functional prediction was performed with WRKY-family proteins. First, they were classified according to functional annotation clustering using DAVID 6.7 software^1^ (Huang et al., 2009). All known and predicted WRKY protein–protein interactions were retrieved from STRING 9.1 software^2^ (Franceschini et al., 2013). The data from both STRING 9.1 and DAVID 6.7 were integrated, analysed and visualized using CYTOSCAPE 3.0.2 software^3^ (Shannon et al., 2003). The WRKYs were broadly categorized into the functional groups of “general transcription” and “defense-regulated transcription factors.”

### *In Vivo* Phosphorylation of WRKY46

For examining phosphorylation of WRKY46 *in vivo*, the *WRKY46* coding sequence was cloned into *pUGW15* (Nakagawa et al., 2007) to generate *p35S-WRKY46* (which expresses an N-terminal HA-tagged WRKY46 under the control of the Cauliflower Mosaic Virus 35S promoter). Transformation of *Arabidopsis* mesophyll protoplasts was performed as described (Yoo et al., 2007; Ranf et al., 2011). After overnight incubation in the dark at 22°C, protoplasts were treated with either water or 100 nM flg22 for 15 min. Protoplasts were harvested by centrifugation and immediately frozen in liquid nitrogen. Dephosphorylation assays were performed as described (Maldonado-Bonilla et al., 2014). Briefly, lambda phosphatase (400 units; Upstate-Millipore) was added to the protoplast extracts for 1 h to dephosphorylate the proteins. After SDS-PAGE, the proteins were transferred to a nitrocellulose membrane (Macherey and Nagel) and immunoblotted against anti-HA.11 antibody (Eurogentec).

### *In Vivo* Stability of WRKY46

To check the stability of WRKY46 after PAMP elicitation, protoplasts were transfected with *p35S-WRKY46* constructs as described above. Protoplasts were treated with 2.5 μM cycloheximide to stop the translation and immediately elicited with 100 nM flg22. Samples were harvested by centrifugation at the indicated time points and were subjected to immunoblotting with anti-HA11 antibody to check the levels of WRKY46 proteins.

### Promoter-Luciferase (LUC) Assay

The promoter-LUC assay was performed as described earlier (Ranf et al., 2011). Briefly, protoplasts were isolated and co-transformed with three vectors: an effector construct for *WRKY46* overexpression, *pNHL10-LUC* (reporter construct) and *pUBQ10-GUS* (normalization construct). After overnight incubation in the dark at 22°C, 200 μM luciferin were added and mixed gently by inverting the tubes. Then, 100 μL of protoplasts were transferred into 96-well microtiter plates suitable for luminescence measurements (Greiner) and were elicited with 100 nM flg22/elf18, 200 μg/mL chitin or water as control. The luciferase activity kinetics were measured for 3 h with a 96-well plate luminescence reader (Luminoscan, Thermo Scientific). After the measurement, protoplast samples were directly lysed by adding 10 μL of 10-fold concentrated GUS extraction buffer (final concentration: 50 mM NaPO_4_ pH 7.0, 1 mM EDTA, 0.1% Triton X-100, 10 mM beta-mercaptoethanol) and vortexing. GUS-activity was measured upon incubation with 4-methylumbelliferyl glucuronide (4-MUG; 15 min at 37°C), based on the fluorometric detection of the reaction product 4-methyl umbelliferone (4-MU). Values are expressed as LUC/GUS ratios relative to the CFP-transfected controls (at timepoint 0 min).

### Localization of WRKY46

*WRKY46* was cloned into *pEXSG-YFP* vector (Feys et al., 2005) to express WRKY46 with a C-terminal YFP-tag. ERF104-CFP (Bethke et al., 2009) was used as a nuclear marker. Protoplasts were isolated, transfected with *WRKY46-YFP* with or without *ERF104-CFP* constructs, incubated overnight and observed under a laser scanning confocal microscope (LSM 710 Laser Scanning System; Carl Zeiss) as described Reddy et al. (2014). For YFP detection, excitation wavelength, and emission filters were 514 nm/band-pass 520–540 nm. For CFP detection, excitation wavelength and emission filters were 458 nm/band-pass 465–500 nm. Chloroplast auto-fluorescence was recorded using band-pass 650–710 nm settings.

### Statistical Analyses

All statistical analyses were performed using GraphPad Prism 5^4^.

## Results

### *Arabidopsis* MPK3 Phosphorylates WRKY46 *In Vitro*

**FIGURE 1:**
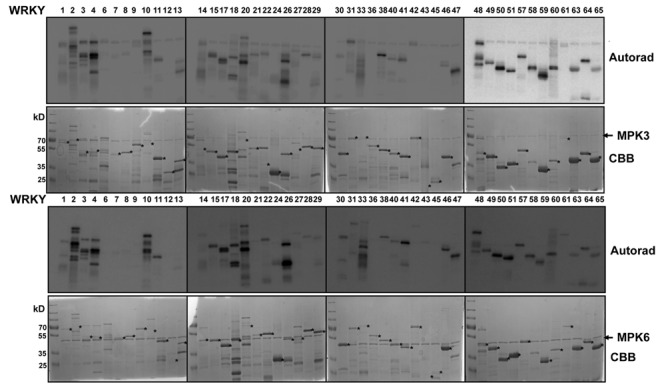
***In vitro* phosphorylation of *Arabidopsis* WRKYs by MPK3 and MPK6.** Forty-eight *Arabidopsis* WRKY transcription factors were expressed in bacteria as His-tagged recombinant proteins, purified and used as substrates for *in vitro* phosphorylation by GST-tagged MPK3 **(Upper)** or untagged MPK6 proteins **(Lower)**. Expected sizes of the WRKYs are marked with asterisks. Note that truncated products or aberrant migration patterns in SDS-PAGE, as seen in the Coomassie Brilliant Blue staining (CBB), are seen for several WRKYs. The experiment was repeated twice with similar results.

We expressed 48 members out of the 74 WRKYs, covering all three WRKY groups, as recombinant proteins. MPK3 and MPK6 were also prepared and activated by incubating with a constitutively active MAPKK (abbreviated as MKK5^DD^; Lee et al., 2004). *In vitro* kinase assays were performed using the 48 purified WRKY proteins as substrates for MPK3 or MPK6 (**Figure 1**). Most of the WRKYs (or their truncated products) were phosphorylated by both kinases. The WRKYs showed differential phosphorylation intensities, possibly reflecting either the strength of their interaction with MPK3/MPK6 or the number of putative MAPK-targeted (SP/TP) sites present in them.

**FIGURE 2:**
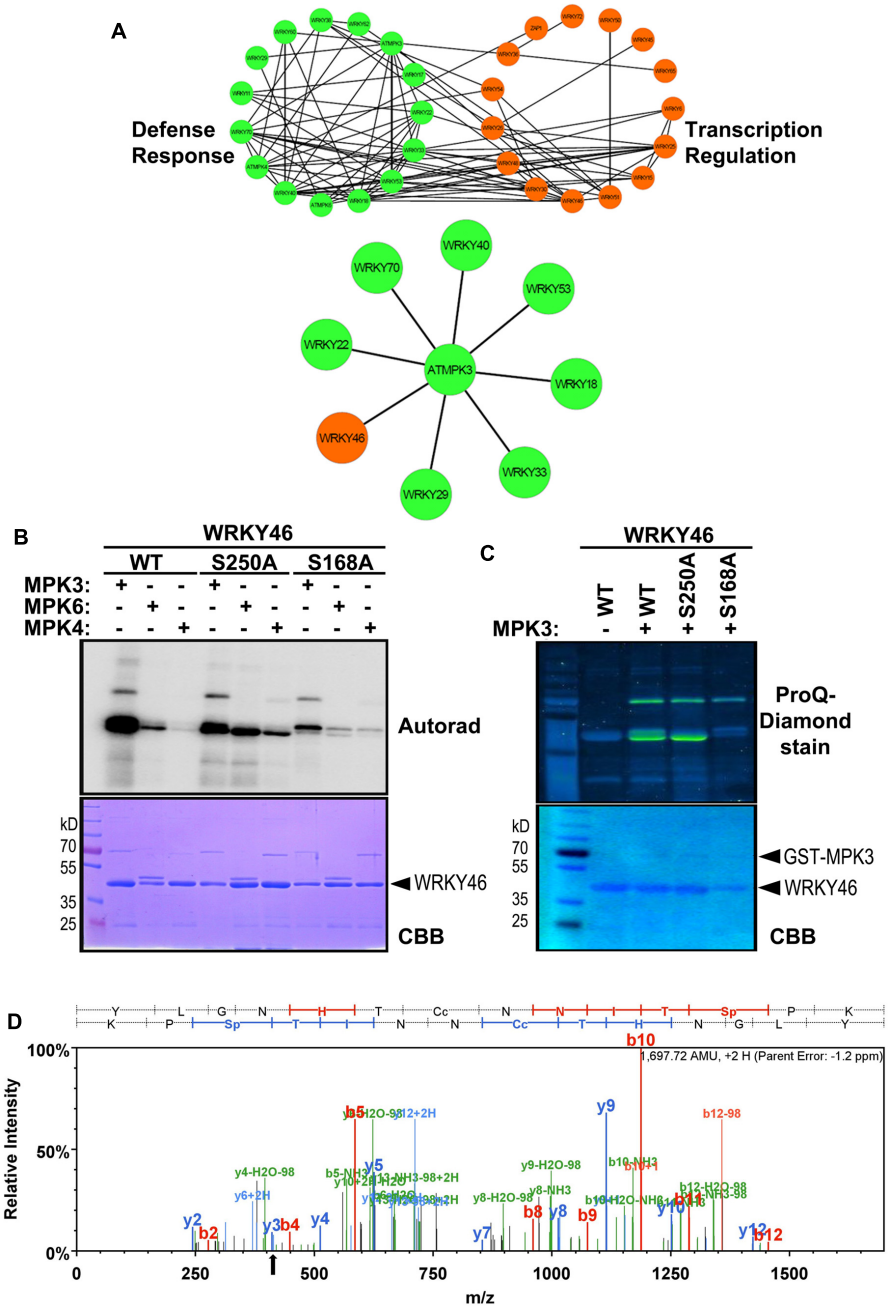
**WRKY46 is a potential defense-related phosphorylation target of MPK3. (A)** The functional annotation of all WRKYs was performed using DAVID 6.7 tool dividing all WRKYs into two groups (defense response, DR, or transcriptional regulation, TR; top network). The known and the predicted protein-protein interactions were analyzed by STRING 9.1 tool. Finally, all the information was integrated, analyzed and visualized using CYTOSCAPE 3.0.2 program. The (bottom) network shows WRKY46 as a potential defense-related MPK3 target along with many known MPK3 interaction partners. **(B)** *In vitro* phosphorylation of WRKY46 by MAPKs and phospho-site mapping. His-tagged WRKY46 and its phospho-site variants (S168A and S250A) were expressed using bacterial expression systems and used as substrates for phosphorylation by GST-MPK3, GST-MPK4, and tag-free MPK6 proteins, which were pre-activated with constitutively active MKK5^DD^. The proteins from the *in vitro* kinase assays were separated by SDS-PAGE and phosphorylation visualized by autoradiography (Autorad). Note that the MAPK bands can be seen above the WRKY46 proteins (GST-MPK3 or -MPK4 = ∼70 kD; MPK6 = ∼48 kD) The experiment was repeated twice with similar results. **(C)** Phosphorylation of WRKY46 and its variants by MPK3 was performed as described in (**B**; but using non-radioactive ATP). ProQ diamond^®^ staining was used to visualize phosphorylated proteins. CBB of the gels was performed as loading control. **(D)** Mass spectrometry (MS) analysis of WRKY46. The phosphorylated WRKY46 bands (from **C**) were excised from the gels, trypsin digested and analyzed by LC-MS/MS. A phospho-peptide demonstrating phosphorylation of WRKY46 at position S168 (as reflected by y3 peak at 411 m/z) is shown.

To narrow down the candidate WRKYs possibly involved in plant defense responses, *in silico* data-mining for protein-protein interaction (STRING 9.1, Franceschini et al., 2013) and functional annotation of roles in defense or transcriptional regulation (DAVID 6.7, Huang et al., 2009) was carried out with the WRKYs and MAPKs used in this study. A network of at least eight WRKYs, including WRKY46, was linked to MPK3 and may be involved in plant defense responses (**Figure 2A**).

*Arabidopsis* WRKY46 is a group III WRKY of 295 amino acids (molecular weight of 33.6 kDa) and contains two consensus MAPK phosphorylation sites at positions S168 and S250. We performed site-directed mutagenesis to change these two sites from Ser to Ala. To determine which phosphorylation site of WRKY46 can be phosphorylated by MAPKs *in vitro*, His-tagged recombinant WRKY46 protein variants were purified and used for MAPK phosphorylation assays. Using MPK3, MPK4 and MPK6 with comparable kinase activities (estimated by kinase assays with artificial myelin-basic protein as substrate), MPK3 showed the strongest phosphorylation of WRKY46 (**Figure 2B**). The observation was also confirmed by using a non-radioactive kinase assay with MPK3 and subsequent Pro-Q diamond phosphoprotein staining (**Figure 2C**). The MPK3-phosphorylated WRKY46 bands were excised from the non-radioactive gel, trypsin-digested and subjected to mass spectrometry (MS) analysis. A phospho-peptide containing phosphorylated S168 was detected (**Figure 2D**). Phosphorylation at S250, however, cannot be excluded since the peptide coverage of the MS analysis did not include this region of the protein. Nevertheless, MPK3-mediated phosphorylation of the WRKY46^S168A^ variant was strongly reduced compared to WRKY46^S250A^ (**Figures 2B,C**), so that S168 may be the preferred *in vitro* phospho-site. The recombinant WRKY46^S168A,S250A^ double mutant was, unfortunately, poorly expressed in bacteria and it was difficult to purify sufficient amounts for comparison in the *in vitro* kinase assay. Curiously, the phosphorylated WRKY46 band(s) often show a double band, except for the WRKY46^S250A^ variant; this may mean that phosphorylation at S250 results in a mobility shift in SDS-PAGE (**Figures 2B,C**).

### *In Vivo* Phosphorylation of WRKY46

**FIGURE 3:**
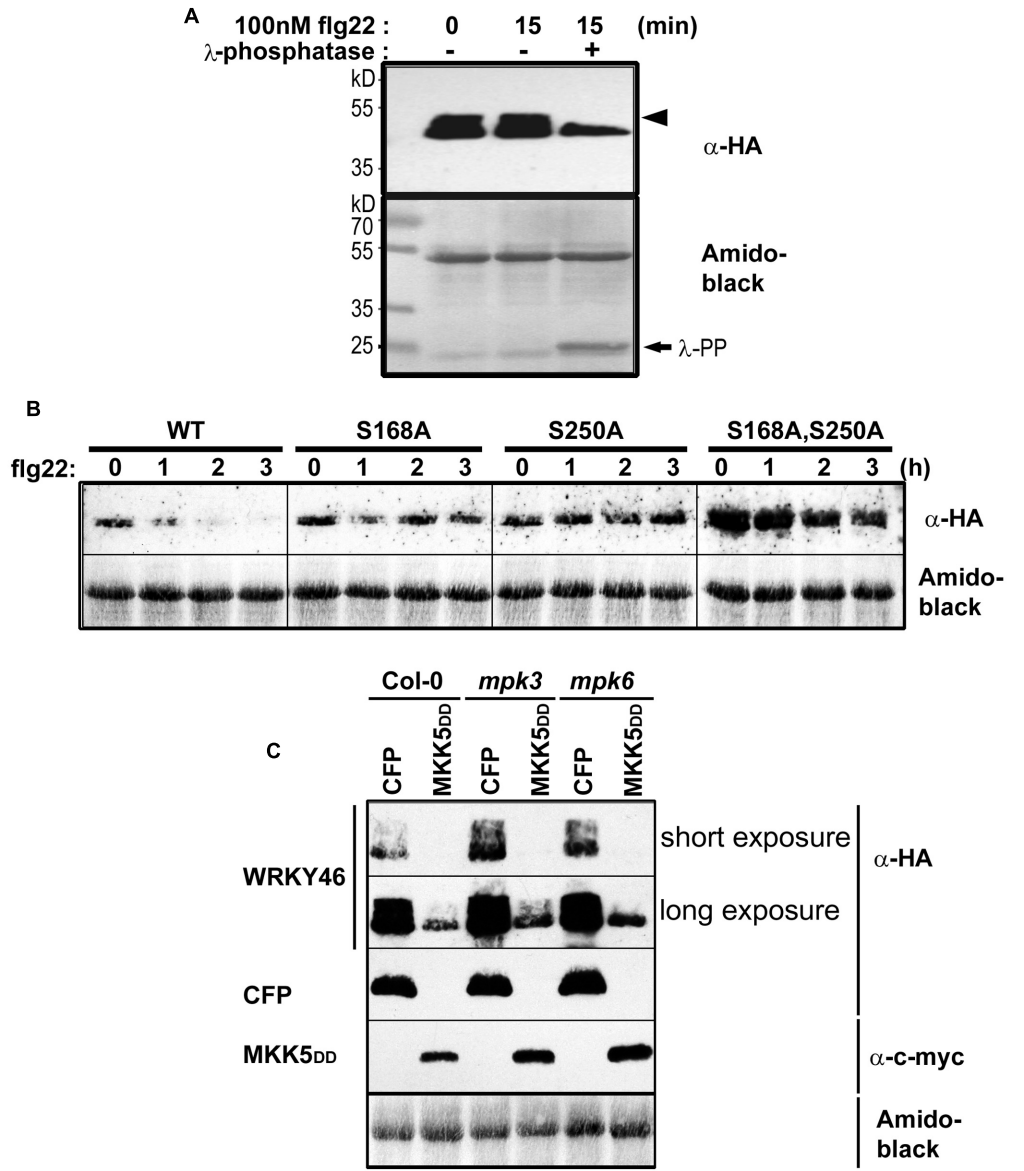
***In vivo* phosphorylation and alteration in WRKY46 stability. (A)** *Arabidopsis* mesophyll protoplasts were transfected with *p35S-WRKY46* (expressing an HA-tagged protein). After overnight incubation, protoplasts were elicited with 100 nM flg22 for 15 min. Total protein extracts were incubated with or without lambda phosphatase (λ-PP) for 1 h (37°C), separated by SDS-PAGE and western blot analysis was performed using anti-HA antibodies. Amido black staining was used to monitor equal loading of the proteins. The experiment was performed twice. Note a mobility shift of WRKY46 (indicative of phosphorylation) is marked by an arrowhead. **(B)** *Arabidopsis* mesophyll protoplasts expressing different WRKY46 variants were simultaneously treated with 100 nM flg22 and 2.5 μM cycloheximide (to block translation) and harvested at the time points indicated. The protein levels of WRKY46 variants were estimated by immunoblotting using anti-HA antibody. Amido black staining (showing the Rubisco large subunit band) was performed to monitor equal loading of proteins. The experiment was repeated at least five times. Note that the gels were blotted on the same membrane to allow direct comparison of band intensities. **(C)** *Arabidopsis* mesophyll protoplasts derived from the indicated genotype were co-transfected with *p35S-WRKY46* and either *p35S-mycMKK5^DD^* (expressing a constitutively active MAPK kinase that activates MPK3 and MPK6) or a as control, a *CFP*-expressing plasmid. Immunoblot with the indicated antibodies and loading control monitoring by amido black staining were performed as described above.

To determine whether WRKY46 is phosphorylated *in vivo, WRKY46* was cloned into a vector expressing HA-tagged proteins under control of the Cauliflower Mosaic Virus 35S promoter and transfected into *Arabidopsis* protoplasts. After an overnight incubation (∼15 h) to enable protein expression, protoplasts were treated with flg22 (100 nM) or an equivalent volume of water (as control) for 15 min. Protoplasts were harvested, proteins extracted, and immunoblotted against an anti-HA antibody. An aliquot of proteins from the flg22-treated sample was incubated with lambda phosphatase to dephosphorylate the proteins. As reflected by a mobility shift in SDS-PAGE, which is abolished by the phosphatase treatment, WRKY46 is already phosphorylated before any PAMP elicitation (**Figure 3A**). Since the MAPKs are typically not activated prior to PAMP treatment, it appears that there are other kinase(s) that target WRKY46. As there is no additional phospho-shift after PAMP treatment, it is also not possible to use this assay as an indicator for *in vivo* phosphorylation by PAMP-activated MAPKs, such as MPK3 or MPK6 (see below).

### Phosphorylation of WRKY46 Alters Its *In Vivo* Stability

The half-life of proteins is critical for the regulation of various signaling pathways and stability of several MAPK substrates has been shown to be regulated through phosphorylation after PAMP treatment (Liu and Zhang, 2004; Maldonado-Bonilla et al., 2014; Pecher et al., 2014). Therefore, as an alternative to using phospho-shift in gels, we investigated WRKY46 stability after elicitation of transiently transformed protoplasts with flg22. To facilitate visualization of the reduced protein levels after PAMP addition, cycloheximide was also added to block protein translation. Protoplasts were harvested at different time points after elicitation and subjected to immunoblotting. Reduction in wild type WRKY46 levels was seen 1 h after flg22 treatment, with little-to-no WRKY46 detectable after 2 h (**Figure 3B**). By contrast, flg22-induced reduction in protein levels was less pronounced for the WRKY46^S168A^, WRKY46^S250A^ and WRKY46^S168A,S250A^ mutant variants over the tested period of 3 h. In general, the highest level of protein expression was observed for the WRKY46^S168A,S250A^ double phosphorylation site mutant (**Figure 3B**). Taken together, phosphorylation of WRKY46 (at S168 and/or S250) is required for the flg22-induced degradation *in vivo* and the non-phosphorylatable WRKY46 is more stable.

To directly link this destabilization effect to MAPK activities, we co-expressed MKK5-DD, a constitutively active MAPK kinase that activates MPK3 and MPK6 (Lee et al., 2004, 2015; Lassowskat et al., 2014) together with WRKY46. We also performed these experiments in the *mpk3* or *mpk6* mutant backgrounds to specifically activate only MPK6 or MPK3, respectively. As seen in **Figure 3C**, specific activation of MPK3 and/or MPK6 led to reduced WRKY46 protein levels – thus pointing to a direct effect of MPK3/MPK6-mediated phosphorylation on WRKY46 stability. As *mpk3mpk6* double mutants are embryo-lethal and even the so-called “rescued” mutants have severe growth defects (Wang et al., 2007, 2008), it is difficult to test the effect of knocking-out both MAPKs on WRKY46 stability. The data therefore also suggests functional redundancy between MPK3 and MPK6 in regulating WRKY46 destabilization.

### WRKY46 is a Nuclear Protein and Elevates the Basal Plant Defense Status

**FIGURE 4:**
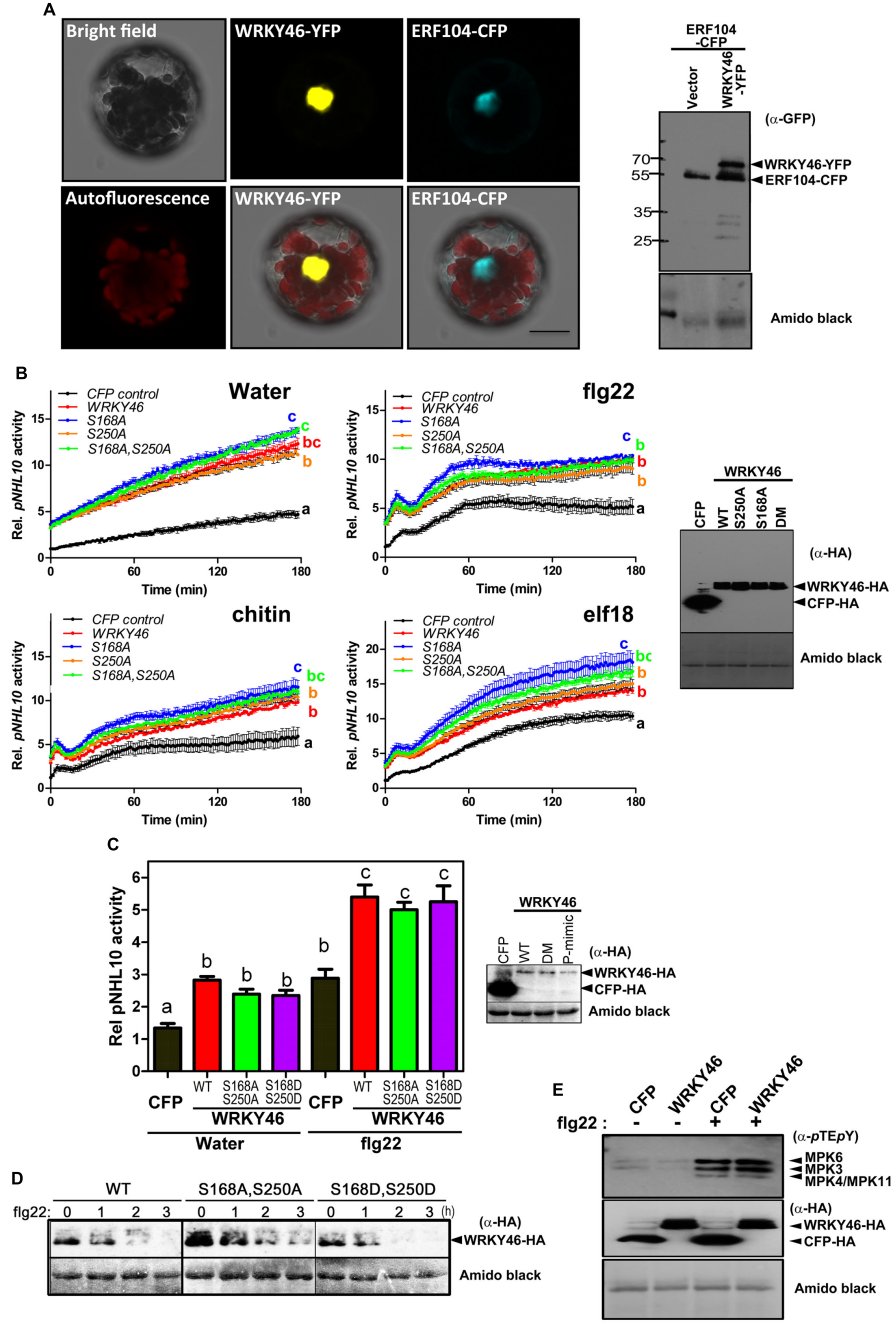
**WRKY46 is nuclear localized and boosts defense-related promoter activity. (A)** *Arabidopsis* Col-0 mesophyll protoplasts were co-transfected with *WRKY46-YFP* and *ERF104-CFP* constructs, with ERF104 serving as a nuclear marker. Confocal laser scanning micrographs of the protoplasts show the nuclear localization of WRKY46. Chlorophyll autofluorescence is also included to visualize chloroplasts. Scale Bar = 20 μm. **(B)** *p35S*-*WRKY46* wild type and mutant variants were transfected into *Arabidopsis* mesophyll protoplasts along with *pNHL10-LUC* and *pUBQ10-GUS* reporter constructs. After overnight incubation, protoplasts were elicited with water, 100 nM flg22/elf18 or 200 μg mL^-1^ crab shell chitin. Luciferase activity was measured for 3 h. After the measurements, GUS-assays were performed with total protein extracts. The data are presented as LUC/GUS ratios relative to the untreated CFP-expressing control (at timepoint 0 min) with error bars indicating standard deviation (SD) of the mean. Different letters indicate statistically significant differences at time point 60 min [two-way repeated measures (RM) ANOVA with Bonferroni post tests, *p* < 0.01]. The experiment was performed three times with similar results (DM = S168A, S250A double phospho-site mutant). **(C)** Experiments after transfection with the indicated *WRKY4*6 variants or *CFP* as a control were conducted as described in **(B)** above and the peak *pNHL10* activities (typically at ∼40 min post elicitation with flg22) plotted. The different alphabets mark statistically distinct groups after one-way ANOVA analysis with Bonferroni’s multiple comparison test. Right panel is the immunoblot analysis of HA-tagged proteins. (P-mimic = S168D, S250D double phospho-mimic mutant). **(D)** WRKY46 protein stability after flg22 treatment was analyzed as described in **Figure 3B**. **(E)** Overexpression of WRKY46 does not alter MAPK activation. *Arabidopsis* mesophyll protoplasts were either transfected with constructs for expressing CFP (as a control) or WRKY46. After overnight incubation, protoplasts were treated with either water or 100 nM flg22 for 10 min. The SDS-PAGE-separated proteins were immunoblotted with anti-*p*TE*p*Y antibody to check for MAPK activation. The experiment was performed twice with similar results. In all cases, equivalent expression of (intact) proteins in the protoplasts was tested by immunoblotting with the indicated antibodies. Amido black staining of the nitrocellulose membranes was used to estimate equal loading (The staining of the Rubisco large subunit is shown).

To examine WRKY46 subcellular localization, we transiently expressed WRKY46-YFP in *Arabidopsis* protoplasts. To visualize the nucleus, protoplasts were co-transfected with a CFP-tagged ERF104 that was previously shown to be nuclear-localized (Bethke et al., 2009). The resulting CFP and YFP fluorescence signals were both in the nuclei (**Figure 4A**). To exclude that WRKY46 might have been tethered to the ERF104 nuclear protein, WRKY46 was expressed alone where the WRKY46-YFP signals were also in the nucleus (**Supplementary Figure S1**). Western blotting using an anti-GFP monoclonal antibody revealed predominantly protein bands with the expected molecular weights for intact WRKY46-YFP and ERF104-CFP fusion proteins (**Figure 4A**), so that the nuclear fluorescence signals detected is not due to free CFP/YFP cleavage products. In addition, we also tested the non-phosphorylatable WRKY46^S168A,S250A^ variant, which is also localized in the nucleus (**Supplementary Figure S1**). These findings are in agreement with WRKY46 functioning as a putative transcription factor in the nucleus (Ding et al., 2014, 2015) and also the increased protein stability of WRKY46^S168A,S250A^ (**Figure 3A**) is not due to its mis-localization.

To investigate the role of WRKY46 in plant defense, we analyzed expression of a luciferase (LUC) reporter driven by a routinely used W-box-containing defense-related promoter, *NDR1/HIN1-like 10* (*NHL10*), in *Arabidopsis* protoplasts co-expressing either different variants of WRKY46 or CFP (as a control for expression of a defense-unrelated protein). Protoplasts were treated with either water, bacterial (flg22/elf18) or fungal (chitin) elicitors and luciferase activity was measured. In protoplasts expressing any WRKY46 variant, an elevated basal promoter activity (time point 0 min) was observed for the water and also the PAMP-treated samples (**Figure 4B**). Also at later time points, all treatments showed an overall elevation of *NHL10* promoter activity in the WRKY46-expressing protoplasts. This suggests that WRKY46 is a positive regulator of the defense-related *NHL10* gene. Interestingly, the S168A variant consistently showed a higher boost on the *NHL10* promoter activity. Since western blotting showed comparable expression of the respective WRKY46 variants (**Figure 4B**, right), the enhanced activity of WRKY46^S168A^ is not simply due to enhanced protein stability/expression (see **Figure 3B**). To further test if phosphorylation of WRKY46 influences its effect on transcription controlled by the *NHL10* promoter, we created a WRKY46^S168D,S250D^ phospho-mimic. For simplicity, we tested only flg22 and concentrated only on the peak *NHL10* promoter activity. As seen in **Figure 4C**, there was no difference in activities of the phospho-mimic (WRKY46^S168D,S250D^) or the non-phosphorylatable (WRKY46^S168A,S250A^) variants compared to wild type WRKY46. However, in line with the role of phosphorylation in regulating WRKY46 stability (compare **Figure 3B**), the phospho-mimic variant is expressed at lower levels (**Figure 4C**). We therefore compared the stability of WRKY46 phosphosite-mutated variants. While trace levels of wild type WRKY46 are expressed 2 h post flg22 treatment, the phospho-mimetic WRKY46 is hardly detectable (**Figure 4D**). Thus, this serves as indirect evidence that mutated residues are functionally mimicking the phosphorylated serines. Taken together, transcriptional activity of WRKY46 does not seem to be directly controlled by its phosphorylation status.

Since the *NHL10* reporter is often used as a reporter for MAPK impact on defense gene expression (Boudsocq et al., 2010), it is possible that there is a feedback regulation on MAPK activities after WRKY46 overexpression. This has been shown for the MAPK substrate, MYC2, which exerts feedback regulation on its phosphorylating MAPK, MPK6, during blue light-induced signaling (Sethi et al., 2014). To investigate whether also WRKY46 affects MAPK activities *in planta*, protoplasts expressing either WRKY46 (or CFP as a control) were treated with water or flg22 for 10 min and MAPK activation was analyzed. Treatment with flg22 leads to strong activation of MPK6, MPK3, and MPK4/11 in both WRKY46 and CFP expressing samples without obvious differences (**Figure 4E**). Thus, WRKY46 does not seem to have feedback regulatory activity on the MAPK pathway; and the enhanced *NHL10* promoter activity is likely due to direct action of the overexpressed WRKY46.

**FIGURE 5:**
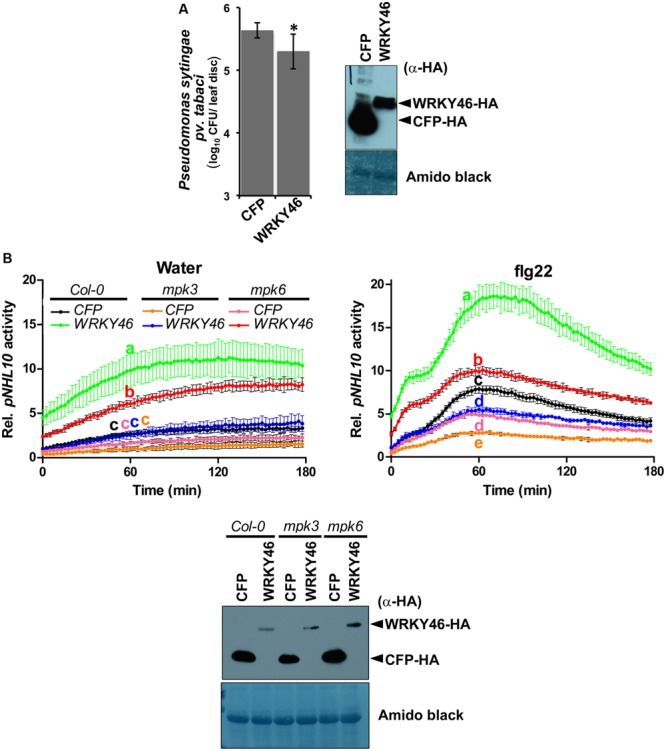
**WRKY46 enhances bacterial resistance and MPK3/6 are required for the WRKY46-mediated boost of *NHL10*-promoter activity. (A)** WRKY46 overexpression slightly enhances resistance against *P. syringae* in tobacco. WRKY46 was transiently expressed in *N. benthamiana* leaves by *Agrobacterium*-mediated delivery. After 48 h, leaves were challenged with *P. syringae* pv. *tabaci*. Colony forming units (CFU) were calculated for samples harvested 2 days post infection. Data shown is the average of three triplicates with error bars indicating standard deviation (^∗^*p* < 0.05; Student’s *t*-test). **(B)** *p35S-WRKY46* was transfected together with *pNHL10-LUC* and *pUBQ10-GUS* constructs into protoplasts derived from *Arabidopsis* Col-0, *mpk3* and *mpk6*, and analyzed as described above **(B)**. Error bars indicate standard deviation. Different letters indicate statistically significant differences at time point 60 min (two-way RM ANOVA with Bonferroni post tests, *p* < 0.01). The experiment was performed three times with similar results. In all cases, protein expression is validated by western blotting as described above.

Transgenic WRKY46-overexpressing *Arabidopsis* plants have been shown to be more resistant to bacterial pathogens (Hu et al., 2012). To validate this, we transiently overexpressed *Arabidopsis* WRKY46 in *Nicotiana benthamiana* leaves by agro-infiltration. After 24 h, the leaves were challenged with *Pseudomonas syringae* pv. *tabaci* and harvested 2 days later. Significantly less bacteria were counted in the leaves overexpressing WRKY46 compared to leaves expressing CFP as a control (**Figure 5A**). Taken together, these observations suggest that WRKY46 is a positive regulator of plant defense.

### MAPKs, and Particularly MPK3, are Required for the Transcriptional Activity of WRKY46

As mentioned above, we could not use the protein mobility phospho-shift in SDS-PAGE to demonstrate an *in vivo* PAMP-activated MAPK phosphorylation of WRKY46, which we routinely use for several other MAPK substrates (Bethke et al., 2009; Maldonado-Bonilla et al., 2014; Pecher et al., 2014). To alternatively address the role of MPK3 and MPK6 in the function of WRKY46, we investigated *NHL10* promoter activities in the respective *mpk* mutants. In protoplasts derived from *mpk3* or *mpk6* plants, a statistically significant reduction of the WRKY46-mediated boost of the *NHL10* promoter activity was observed in comparison to the wild type for both the water (control) or flg22 treatments (**Figure 5B**), indicating that both kinases are important for full WRKY46 activity. Notably, *mpk3* showed a more dramatic effect than the *mpk6* mutant, where the WRKY46-mediated boost on the basal *NHL10* promoter activity was completely abolished (See **Figure 5B** left, compare the green trace to the blue trace). Furthermore, the flg22-activation of *NHL10* promoter was also compromised compared to the CFP control (**Figure 5B**, right, blue and black traces, respectively) in the *mpk3* background. Thus, despite functional redundancy between MPK3 and MPK6 (**Figure 3C**), MPK3 may play a stronger *in vivo* role in regulating the transcriptional activity of WRKY46 (on the *NHL10* promoter). This observation is in agreement to a preferential *in vitro* phosphorylation of WRKY46 by MPK3 than MPK6 (**Figure 2B**).

## Discussion

In order to adapt to new environmental conditions, plants have to reprogram transcription of the appropriate genes. Transcription factors play major roles in the control of gene expression. The WRKY family is among the ten largest families of transcription factors in higher plants and is found throughout the green lineage (green algae and land plants; Rushton et al., 2010). WRKY proteins are involved in growth development, such as embryogenesis (Lagace and Matton, 2004), trichome development (Johnson et al., 2002), senescence (Robatzek and Somssich, 2001), and plant responses to various biotic and abiotic stresses (Cai et al., 2008; Wu et al., 2009). Only a few components of signaling pathways interacting with WRKY transcription factors have been identified. In addition to histone deacetylases and calmodulin (CaM)-binding domain proteins, MAP kinases are one of the major protein classes interacting with WRKY transcription factors (Kim and Zhang, 2004). The ability of WRKY proteins to interact with many other proteins makes it challenging to decipher the entire pathways regulated by them. WRKY proteins can interact among themselves, with VQ-domain proteins, calmodulin-like proteins, chromatin remodeling proteins and most importantly with MAP kinases (Chi et al., 2013). MAP kinase pathways are involved in regulating the activity of WRKY33 and WRKY34 to control the plant defense response and pollen development, respectively (Mao et al., 2011; Guan et al., 2014). It is plausible that the MPK3/6 cascade acts as a molecular hub to integrate different signaling networks of WRKY transcription factors with different upstream cues. This is further substantiated by the fact that almost 70% of the WRKYs are differentially regulated by bacterial pathogens or SA treatment, which also activates the MPK3/6 pathway (Dong et al., 2003).

In the current study, 48 out of 74 WRKY members were identified as *in vitro* phosphorylation substrates of MPK3 and MPK6. *In silico* analysis revealed that WRKY46 may be a putative target of MPK3, which is supported by *in vitro* kinase assays showing a stronger phosphorylation by MPK3 compared to MPK6. MS analysis identified S168 of WRKY46 as a phosphorylation site targeted by MPK3. However, subsequent experiments suggest that S250, the other putative phosphorylation site, is also phosphorylated. Direct *in vivo* MPK3 or MPK6 activation by expressing a constitutively active MKK5 led to destabilization of WRKY46 protein. Thus, MPK3 and/or MPK6 (or perhaps also other unknown kinases activated by these MAPKs) phosphorylate S168, S250 and other positions to regulate its stability. In this respect, a *WRKY46* promoter-based reporter assay revealed transcription activation of *WRKY46* by the calcium-dependent protein kinases, CPK-3, -4, -5, -6, -10, -11, and -30 and several WRKYs were directly phosphorylated by CPKs (Gao et al., 2013).

WRKY46 regulates responses to several abiotic stresses in *Arabidopsis.* For instance, it controls the expression of several genes involved in osmoprotection and redox homeostasis under dehydration stress (Ding et al., 2015). It is also involved in controlling the light-dependent stomatal opening in guard cells (Ding et al., 2014), which may serve as potential entry points for pathogens and therefore contribute to responses to pathogens. Indeed, WRKY46 is also involved in biotic stresses. Similar to the *NHL10* gene shown in this work, over-expression of *WRKY46* in protoplasts was found to increase the expression of endogenous *AVRPPHB SUSCEPTIBLE 3* (*PBS3*) gene, which plays an important role in SA metabolism (van Verk et al., 2011). WRKY46 acts in concert with other WRKYs, including WRKY54 as a positive regulator and WRKY70 as a negative defense regulator, to resist infection by the necrotrophic *Erwinia amylovora* (Moreau et al., 2012). It also coordinates with WRKY53 and WRKY70 to control defense response against the hemi-biotrophic pathogen, *Pseudomonas syringae* (Hu et al., 2012). In general, expression of many WRKY genes is induced by pathogen-related stimuli, possibly in a feedback amplification loop. For instance, the expression of *WRKY46* was highly induced within hours after *P. syringae* and SA treatments (Dong et al., 2003). *WRKY46* transcripts accumulated in protoplasts expressing *avrRpm1, avrB*, or *avrRpt2* in an RPM1- or RPS2- dependent manner, thereby making it an early marker gene in ETI signaling and candidate ETI regulator (Gao et al., 2013). The induction of its expression by PAMPs (**Figure 4B**) also makes it a PTI marker gene. Since WRKY46 can contribute to both PTI and ETI signaling, it is plausible that it mediates interplay between PTI and ETI and this will be an interesting question to address in the future.

The MAPK phosphorylation could control the function of transcription factors or their associated proteins by regulating their *in planta* stability (Pecher et al., 2014; Weyhe et al., 2014). Recently, phosphorylation of LIP5 (LYST-INTERACTING PROTEIN5), a positive regulator of multivesicular body (MVB) biogenesis by MPK6 was found to increase its stability (Wang et al., 2015). Similarly, phosphorylation of *Arabidopsis* ERF6, ACS2, and ACS6 by MPK3/MPK6 increase the stability of these substrates (Li et al., 2012). In case of WRKY46, our data provides evidence that MAPK-mediated phosphorylation upon flg22-treatment controls its stability *in vivo*. The phosphorylation at both S168 and S250 positions seems to be very important for the half-life of the protein, since ablation of phosphorylation at either position leads to increased stability upon elicitation. Hence, the phosphorylation-dependent regulation of protein substrate stability appears to be a common mechanism through which MAPKs regulate plant defense responses.

Localization studies revealed that WRKY46 is localized in the nucleus. This result is consistent with the fact that it acts as a transcription factor, possibly regulating the transcription of defense-related genes. Accordingly, WRKY46 overexpression boosts the defense responsive *NHL10* promoter activity (in the absence of pathogens or PAMPs). Since there are WRKY DNA-binding elements in the *NHL10* promoter (Zheng et al., 2004), which have been shown to be important for regulation of PAMP-induced expression (Pecher et al., 2014), it is likely that WRKY46 acts directly as a positive transcriptional activator *via* such *cis*-elements. The compromised activation of the *NHL10* promoter by WRKY46 overexpression in the *mpk3* or *mpk6* mutants suggests the importance of these two MAPKs, particularly MPK3, in controlling WRKY46 activity. This may seem puzzling for the water-treated samples since no enhanced MAPKs activities are usually detected prior to PAMP elicitation. However, we cannot exclude that some handling stress did, in fact, initiate a transient MAPK activation in the protoplast system and this may trigger a long-lasting effect on the subsequently expressed WRKY46 proteins. This would mean that effect of WRKY46 on the *NHL10* promoter is indirect or at least acts only in synergy with the general stress-induced MAPK activities. In support of the latter, the WRKY46-induction of the *NHL10* promoter in the flg22-treated protoplasts (**Figure 5B**, right) – where MAPKs as well as other signaling pathways are activated – is also compromised by *mpk3* or *mpk6* mutations. In summary, the mode of the WRKY46-MAPKs regulation of *NHL10* promoter is currently unknown but it appears to be unrelated to WRKY46 protein stability or its phospho-status (Note: the phospho-mimic WRKY46 did not show enhanced transcriptional activity). Future experiments could reveal if the MPK3/MPK6 effect may be due to altered affinity of WRKY46 to the promoter or direct impact on its transcriptional activity. Alternatively, a more likely scenario would be that there may be other MPK3/MPK6 targets that influence WRKY46 activity. Furthermore, as already discussed, there may be additional kinase(s) that phosphorylate WRKY46. Likely candidates include members of the calcium-dependent protein kinase family – especially those that have been shown to regulate *WRKY46* expression (Gao et al., 2013). It will be crucial to identify these kinase(s) and understand how they, in concert with MAPKs, control the multiple functions of WRKY46 in developmental processes, abiotic and biotic stress responses.

## Author Contributions

AS and LE designed and performed the experiments shown. PP cloned the WRKYs into the bacterial expression system and screened them for recombinant protein expression. WH performed the LC-MS/MS determination of the phosphorylation sites. DS, AS, and JL supervised the project and all authors contributed to writing the manuscript.

## Conflict of Interest Statement

The authors declare that the research was conducted in the absence of any commercial or financial relationships that could be construed as a potential conflict of interest.

## Footnotes

1 http://david.abcc.ncifcrf.gov/

2 http://string-db.org/

3 http://www.cytoscape.org/

4 www.graphpad.com

## Supplementary Material

Figure S1 **Nuclear localization of WRKY46: **(A)** Confocal laser scanning micrographs of *Arabidopsis* Col-0 mesophyll protoplasts transfected with the indicated *WRKY46-YFP* constructs.** To visualize the nucleus, nuclear DNA was stained with 1 μg mL^-1^ of 4′,6-Diamidino-2-Phenylindole (DAPI) in the presence of 0.05 % Triton-X100 (to permeabilize the membranes). Scale Bar = 10 μm. **(B)** Western blot analysis with α-GFP to show intactness of WRKY46-GFP fusion proteins.
