# Supplementary figures and images for: Regulation of WRKY46 Transcription Factor Function by Mitogen-Activated Protein Kinases in Arabidopsis thaliana
Source: Front Plant Sci. 2016 Feb 4;7:61. doi: 10.3389/fpls.2016.00061 (PMC4740394; doi:10.3389/fpls.2016.00061)

**A**WRKY46  
WTWRKY46  
S168A S250Achlorophyll  
autofluorescence

YFP

DAPI

bright field

merged

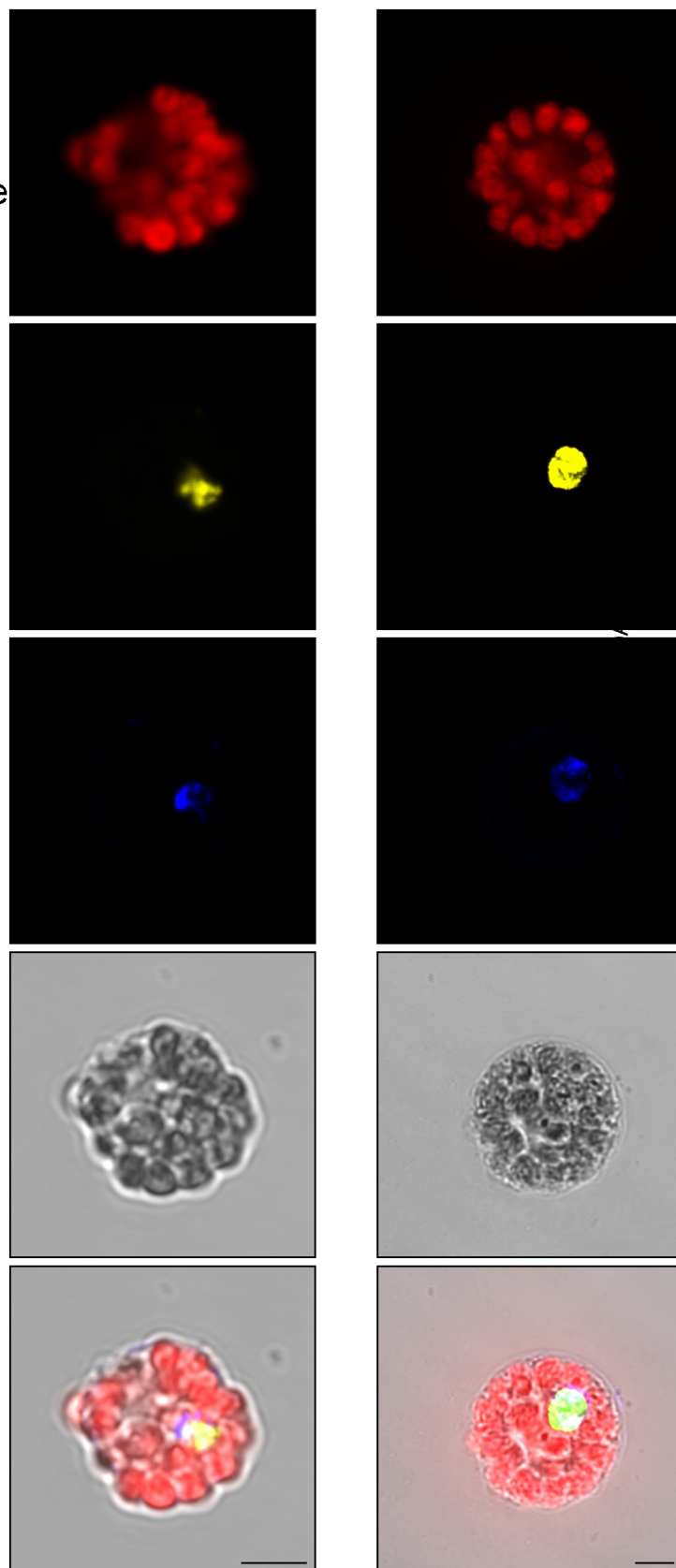**B**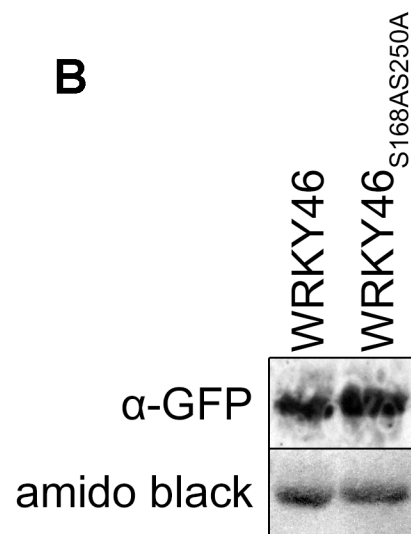bar = 10  $\mu$ m

Supplement: Figure S1 — Nuclear localization of WRKY46: (A) Confocal laser scanning micrographs of Arabidopsis Col-0 mesophyll protoplasts transfected with the indicated WRKY46-YFP constructs. To visualize the nucleus, nuclear DNA was stained with 1 μg mL-1 of 4′,6-Diamidino-2-Phenylindole (DAPI) in the presence of 0.05 % Triton-X100 (to permeabilize the membranes). Scale Bar = 10 μm. (B) Western blot analysis with α-GFP to show intactness of WRKY46-GFP fusion proteins. [file Image_1.PDF]
